# Rural–urban residence in relation to cannabis use influences and behaviors among a sample of US young adults reporting past-month cannabis use

**DOI:** 10.1111/ajad.70185

**Published:** 2026-07-01

**Authors:** Katelyn F. Romm, Amanda Y. Kong, Patricia A. Cavazos-Rehg, Carla J. Berg

**Affiliations:** 1 TSET Health Promotion Research Center, Stephenson Cancer Center, University of Oklahoma Health Campus, Oklahoma City, Oklahoma, USA; 2 Department of Pediatrics, College of Medicine, University of Oklahoma Health Campus, Oklahoma City, Oklahoma, USA; 3 Division of Public Health Sciences, Department of Social Sciences and Health Policy, Wake Forest University School of Medicine, Winston-Salem, North Carolina, USA; 4 Department of Psychiatry, Washington University School of Medicine, St. Louis, Missouri, USA; 5 Department of Prevention and Community Health, Milken Institute School of Public Health, George Washington University, Washington, DC, USA; 6 George Washington Cancer Center, George Washington University, Washington, DC, USA

## Abstract

**Background and Objectives:**

Despite growing cannabis use rates among US rural-residing young adults, less research has examined associations of rural–urban residence with cannabis use influences (e.g., harm perceptions, peer use) or behaviors (e.g., use frequency, driving under the influence of cannabis [DUIC]).

**Methods:**

Using 2023 online survey data from 1961 US young adults (aged 18–34) reporting past-month cannabis use (*M*_age_ = 26.86 [standard deviation = 4.61], 14.7% rural-residing, 38.2% racial/ethnic minority, 59.2% female), multivariable regressions controlling for sociodemographics and state non-medical cannabis legalization examined associations of (1) rural–urban residence with cannabis use influences (motives, perceptions, mental/physical health, parent/peer use, advertising exposure); and (2) rural–urban residence and use influences with use behaviors (past-month days of use, past 6-month DUIC, consequences).

**Results:**

Rural (vs. urban) residence was associated with three use influences (i.e., greater coping motives, lower perceived harm of cannabis use, higher odds of a mental health diagnosis), more frequent cannabis use and DUIC, but fewer use consequences. All three influences were associated with more frequent use and having a mental health diagnosis was associated with more frequent DUIC. Greater coping motives and lower harm perceptions were associated with fewer consequences, and having a mental health diagnosis was associated with greater consequences.

**Discussion and Conclusions:**

Rural (vs. urban) young adults reported more frequent cannabis use and DUIC, which may be associated with low cannabis-related harm perceptions and using cannabis to cope with stress/mental health symptoms.

**Scientific Significance:**

Interventions targeting rural young adults that address mental health symptoms and risk perceptions, including DUIC-related risks, may be needed.

## 1 ∣ INTRODUCTION

Cannabis is the most commonly used federally illicit substance among US young adults (aged 18–34).^1^ US young adults’ past-month cannabis use rates have increased from 19.6% in 2014 to 24.1% in 2024,^1^ alongside increases in cannabis legalization for medical and non-medical (i.e., recreational) purposes. Despite some research suggesting associations of cannabis use with improvements in certain self-reported health outcomes (e.g., chronic pain, chemotherapy-induced nausea),^2^ more frequent use during young adulthood is associated with adverse health (e.g., respiratory disorders, cardiovascular disease) and behavioral outcomes (e.g., cannabis use disorder [CUD], driving under the influence of cannabis [DUIC]).^3^

Historically, cannabis use was more common among urban, relative to rural-residing, US young adults.^4^ However, rates of past-month use among rural young adults are steadily rising, with National Survey on Drug Use and Health 2023 data suggesting past-month rates of 25.1% and 26.1% among rural and urban young adults, respectively.^5^ Moreover, a growing body of research indicates that rural (vs. urban) young adults report more frequent cannabis use and greater use-related consequences (e.g., hazardous use, DUIC).^6,7^

Individual-, interpersonal-, and contextual-level factors predict young adults’ cannabis use outcomes. At the individual level, cannabis use motives (e.g., to cope with stress, make activities more enjoyable), perceptions (e.g., low perceived impact of cannabis use on harm to health), and both mental (e.g., mood/anxiety disorders) and physical health conditions are associated with more frequent use and greater use consequences (e.g., CUD, DUIC).^8–11^ At the interpersonal and contextual levels, those with greater exposure to cannabis use in their social networks (i.e., parents, peers) and via advertising (e.g., via billboards, online) experience worse cannabis-related outcomes.^9,12,13^

Rural young adults have unique experiences that may be associated with higher levels of these multilevel factors. For instance, rural (vs. urban) individuals report lower substance use (i.e., tobacco use; aggregated alcohol, tobacco, cannabis, illicit drug use) risk perceptions,^14,15^ and indicate that DUIC is safer than DUI of other substances and may even improve driving ability.^16^ Moreover, rural (vs. urban) individuals report greater mental and physical health symptoms,^17^ less access to health services to treat these symptoms,^18^ and greater boredom.^19^ Thus, rural young adults may report greater cannabis use motives related to coping with mental and/or physical health symptoms and seeking enjoyment.

At the interpersonal level, rural young adults may experience greater substance use exposure and more permissive use norms from both parents and peers. Parents in rural (vs. urban) areas are more likely to use substances in the home, and rural youth are more likely to receive alcohol from parents.^20,21^ Although less research has examined geographic differences in parental cannabis use, research suggests stronger substance use norms within rural, relative to urban families.^22^ With regard to peers, young people in rural (vs. urban) areas more frequently engage in substance use with peers due to fewer alternative entertainment options.^20,21^

Finally, at the contextual level, rural young adults may experience some exposure to cannabis advertising, as cannabis marketing is common in states with both legalized non-medical and medical (many of which have high rural population density) cannabis.^13,23^ For instance, medical cannabis dispensaries in rural Oklahoma largely presented as retail locations and engaged in advertising of specific products and price promotions,^23^ with 75% of adults reporting past-month cannabis marketing exposure (>60% reporting outdoor marketing exposure).^13^ Also common is exposure to cannabis advertising/marketing online, particularly on social media.^13^

The current study identified associations of rural–urban residence with previously-documented multilevel influences of cannabis use (i.e., motives, perceptions, mental/physical health diagnoses, parent use, peer use, advertising exposure), as well as rural–urban residence and use influences with cannabis use behaviors (i.e., past-month use frequency, DUIC, use consequences). Based on the aforementioned literature, which indicates lower substance use risk perceptions,^14–16^ greater mental/physical health symptoms,^17,18^ boredom,^19^ more permissive parental substance use attitudes,^20–22^ and greater substance use with peers^21,22^ among young people in rural (vs. urban) communities, it was hypothesized that rural (vs. urban) young adults would report stronger cannabis-related coping and enjoyment motives, lower perceived addictiveness and harm, greater peer use, as well as greater likelihood of mental/physical health diagnoses and parent use. These cannabis use influences were, in turn, hypothesized to be associated with greater cannabis use frequency, DUIC, and use consequences.^8–12^ Although we did not expect rural–urban differences in cannabis advertising exposure, advertising exposure was hypothesized to be associated with greater cannabis use frequency, DUIC, and consequences.^13^ Findings from this study may inform public health prevention and intervention efforts aimed at reducing problematic cannabis use among young adults, particularly those in rural areas.

## 2 ∣ METHODS

### 2.1 ∣ Participants and procedures

The current study analyzed baseline survey data among young adults (aged 18–34) in the *Ca*nnabis *R*egulation, *M*arketing, & *A*ppeal (CARMA) study, which launched in June–November 2023, involves survey assessments every 6 months for 2 years to examine socio-contextual correlates of cannabis use, and was approved by the George Washington University Institutional Review Board.

Study ads were posted on Facebook and targeted individuals ages 18–34 who were US residents and English-speaking. Purposive, quota-based sampling ensured ~50% of the sample reported current cannabis use, ~50% male/female sex, and ~40% identified as racial and/or ethnic minority. After clicking an ad, individuals were sent a message via chatbot on Facebook Messenger with a study description and screening questions (e.g., age, race/ethnicity, sex, cannabis use). Those deemed preliminarily eligible were sent a link to the study description and consent form in Alchemer (an online survey platform), screened to confirm eligibility, and administered the baseline survey. Participants confirmed participation in the study a week later by responding to an emailed survey link. Fraud prevention efforts included the use of the chatbot prescreening to ensure each individual had a Facebook account, withholding details of eligibility criteria before screening, examining data validity (e.g., duplicate IP addresses, survey completion time), and confirming contact information before providing incentives.

Of the 18,426 Facebook profiles who clicked ads, 6908 (37.5%) completed the pre-screening, and 6128 (88.7% of those who completed the pre-screening) were preliminarily eligible and provided screener and confirmation links. Of the 5081 (82.9%) who consented, 129 (2.2%) were excluded due to (a) ineligibility (*n* = 14) or (b) not completing the screening (*n* = 115). Among the remaining 5672 individuals, 974 (17.2%) provided only partial survey data. Of the 4698 who completed the baseline survey, 313 (6.7%) were not sent confirmation links because they did not provide valid contact information. Of the 4385 provided confirmation links, 4031 (91.9%) confirmed participation, were fully enrolled, and received a $10 Amazon e-gift card incentive (further detailed in prior work^24^). Preliminary analyses assessing rural–urban residence in relation to past-month cannabis use status (i.e., any vs. no use) suggested no significant differences (Pearson *χ*^2^ = 3.57, *p* = .060). Because we focus on mechanisms of use outcomes among those who use cannabis, current analyses include those reporting past-month cannabis use (*N* = 1961).

### 2.2 ∣ Measures

#### 2.2.1 ∣ Sample selection variable: Past-month cannabis use

Participants were asked, “In the past 30 days, how many days did you use marijuana (also known as cannabis, pot, weed, hash, kush)?” with response options of 0–30 days. Those who reported ≥1 day of past-month use were included in the current analyses.

#### 2.2.2 ∣ Rural–urban residence

Participants were asked, “What is your current zip code of residence (i.e., zip code where you currently live; If you attend college and live on campus, please provide your campus zip code)?” The Department of Agriculture Rural–Urban Commuting Area (RUCA) codes categorized participants’ zip codes as “rural” (RUCA code of large rural town, small rural town, or isolated rural town) or “urban” (RUCA code of metropolitan).^25^

#### 2.2.3 ∣ Cannabis use influences

*Cannabis use motives* were assessed with 6 items from the Marijuana Decisional Balance Scale,^26^ asking participants to “Consider the reasons you currently use marijuana. Rate the extent to which each item may impact your decision” (1 = Not at all to 5 = Very much). Exploratory factor analysis yielded a two-factor solution consisting of coping (four items; relieve stress, help me sleep, relieve pain, manage nausea; *α* = .92) and enjoyment motives (two items; create opportunities for social activities, make everyday activities more enjoyable, *α* = .91). Items for each factor were averaged, with higher scores indicating higher motives. *Cannabis use perceptions* were assessed by asking participants, “How addictive do you think marijuana is?” and “How harmful to your health do you think the use of marijuana is?” with response options from 1 = Not at all to 7 = Extremely.

*Mental and physical health diagnoses* were assessed by asking participants to select whether they have ever been diagnosed with any of the following mental (i.e., depression, anxiety disorder, other mood disorder, attention deficit disorder, learning-related condition, substance use problem) and physical (i.e., cancer, pain disorder, seizure disorder, other significant physical health condition) health conditions. Those reporting ≥1 mental and physical health diagnosis were classified as having a mental and physical health condition, respectively.

Regarding *peer cannabis use*, participants were asked, “Out of your 5 closest friends, how many of them use marijuana (in any form)?” with response options from 0 to 5 or more. *Parent cannabis use* was assessed by asking participants, “Do any of your parental figures use marijuana (in any form)?” with response options of yes or no. *Advertising exposure* was assessed by asking participants, “In the past 6 months, how often have you noticed marijuana being advertised or promoted in various places (i.e., stores/kiosks, online, billboards, TV/radio, newspapers/magazines, direct communication) with response options of 0 = Not at all to 5 = More than once a day. Responses were aggregated across sources, with higher scores indicating more frequent advertising exposure (*α* = .85).

#### 2.2.4 ∣ Cannabis use behaviors

*Frequency of past-month cannabis use* was assessed as described above (1–30 days). *DUIC* was assessed by asking participants, “During the past 6 months, how many times did you drive a car or vehicle when you had been using marijuana” (0 = 0 times, 1 = 1 time, 2 = 2–3 times, 3 = 4–5 times, 4 = 6+ times). *Cannabis use consequences* were assessed with 8 items asking participants, “Consider your prior experiences using marijuana. Rate the extent to which each item has impacted you. Using marijuana has…(made me less active/energetic; impaired my judgment, made me endanger myself/others, do things I regret; gotten me in trouble with the law; made me feel bad physically; made people who are important to me disapprove of me; reduced my ability to pay attention or remember things; made me have unpleasant psychological effects; made me neglect obligations to family, work, or school)” on a scale from 0 = Not at all to 5 = Very much.^27^ Exploratory factor analysis yielded a one-factor solution; items were averaged with higher scores indicating greater consequences (*α* = .87).

##### 2.2.4.1 ∣ Descriptive use characteristics

To further characterize participants’ cannabis use, we assessed self-reported distance to nearest cannabis retailer (“How long would it take you to get to the nearest marijuana shop using your usual mode of transportation?”; 1 = Less than 5 min to 8 = More than 1 h), most common mode of cannabis use (“How do you use marijuana most of the time?”) with response options of dried herb (smoked or vaped, including joints, bowls, waterpipes); oils (cannabis oils or liquids for vaping, cannabis oils or liquids taken orally, tinctures); edibles; and concentrates/other (concentrates, hash or kief, topical, other), and use of cannabis for medical or recreational purposes (“Currently, do you use marijuana for medical or recreational purposes?”) with response options of only medical, primarily medical (medical), only recreational, and primarily recreational (recreational) purposes.

#### 2.2.5 ∣ Covariates

Covariates included self-reported age, sex (male, female), race (White, Black, Asian, another race), and ethnicity (Hispanic, non-Hispanic). Participants indicated their state of residence, which was used to determine whether they lived in a state with legal non-medical cannabis (yes, no).^28^

### 2.3 ∣ Data analysis

**FIGURE 1:**
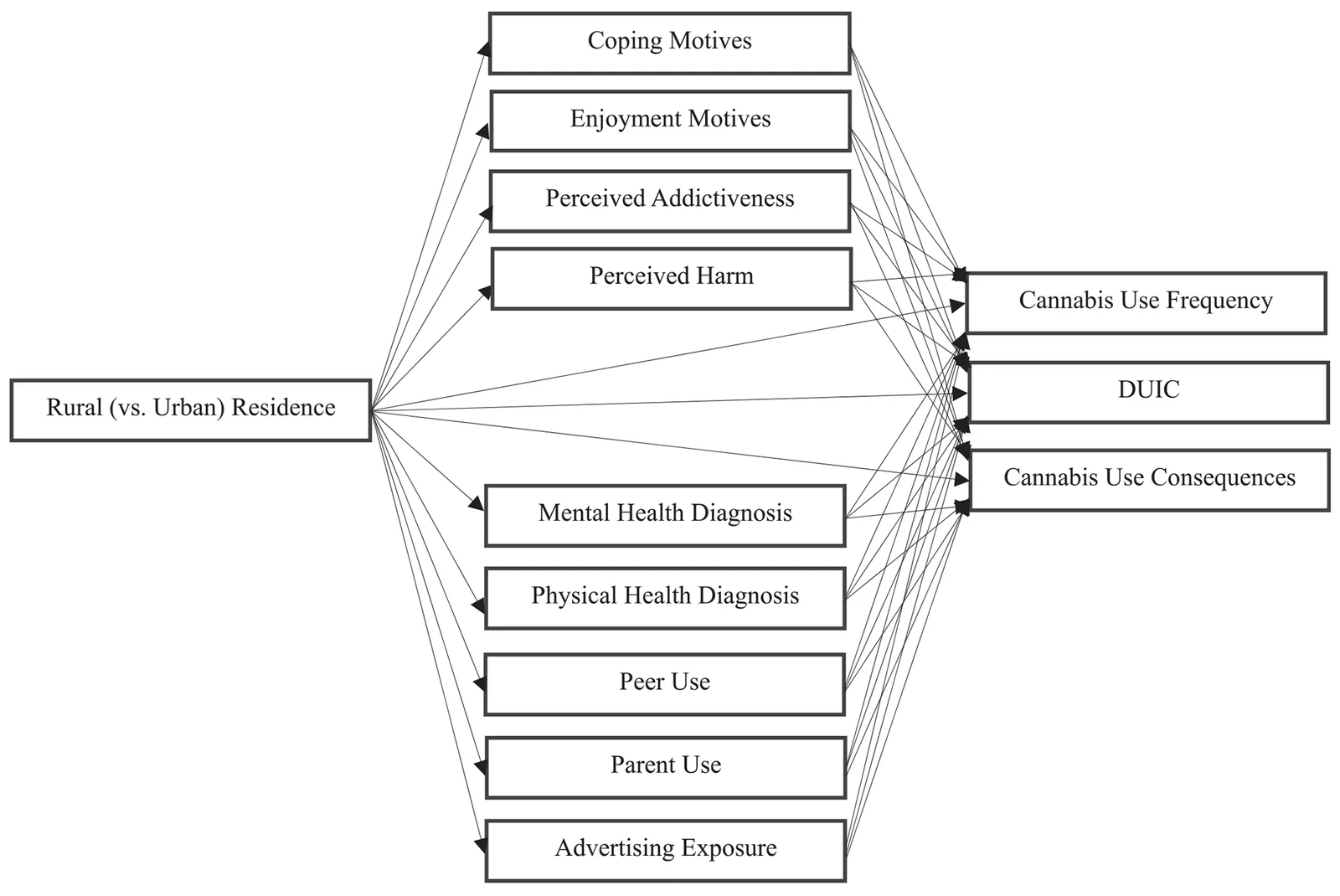
Conceptual model depicting associations of rural–urban residence with cannabis use influences (i.e., motives, perceptions, mental/physical health diagnoses, peer use, parent use, advertising exposure) and behaviors (i.e., cannabis use frequency, driving under the influence of cannabis [DUIC], cannabis use consequences) and associations of cannabis use influences with behaviors.

Descriptive analyses characterized participants and examined response distributions. Bivariate analyses (*χ*^2^ tests, independent samples *t* tests, Pearson’s correlations, one-way analyses of variance) examined associations of covariates and rural–urban residence with cannabis use influences (i.e., motives, perceptions, mental/physical health diagnoses, peer/parent use, advertising exposure) and behaviors (i.e., use frequency, DUIC, use consequences) and associations of cannabis influences with behaviors. Given limited published data on cannabis use characteristics for young adults residing in rural versus urban areas, we conducted supplemental analyses to examine associations of rural–urban residence with state non-medical cannabis legalization, distance to nearest cannabis retailer(s), mode of cannabis use, and use for medical versus recreational purposes. Multivariable regressions first examined rural–urban residence in relation to cannabis use influences. Next, hierarchical regressions examined rural–urban residence (Step 1) and cannabis use influences (Step 2) in relation to cannabis use behaviors (see conceptual model in Figure 1). Supplemental analyses examined cannabis use influences in relation to cannabis use behaviors, excluding rural–urban residence from the model. Analyses were conducted in SPSS v30 and controlled for all covariates.

## 3 ∣ RESULTS

### 3.1 ∣ Participant characteristics

Shown in Table 1, participants were 26.86 years old on average (standard deviation [SD] = 4.61); 50.7% resided in a state with legal non-medical cannabis; 59.2% were female; 61.8% identified as White; 21.6% identified as Hispanic; and 14.7% resided in a rural area. Participants reported high coping (*M* = 4.52, SD = 0.85) and enjoyment (*M* = 4.05, SD = 1.11) motives (range: 1–5), moderate addictiveness (*M* = 3.71, SD = 1.95) and low harm (*M* = 2.77, SD = 1.66) perceptions (range: 1–7), an average of 3–4 (*M* = 3.12, SD = 1.53) friends who use cannabis, and infrequent cannabis advertising exposure (*M* = 1.44, SD = 1.13, range: 1–5). Additionally, 68.7% and 16.3% reported a mental and physical health diagnosis, respectively, and 39.6% reported parental cannabis use. Regarding cannabis use outcomes, participants reported using cannabis 14.19 (SD = 11.30) of the past 30 days, infrequent DUIC (*M* = 0.91, SD = 1.42, range: 0–4), and low use consequences (*M* = 1.99, SD = 0.86, range: 1–5). See Table S1 for further details regarding the geographic breakdown of the sample.

### 3.2 ∣ Associations of rural–urban residence with cannabis use influences

Bivariate analyses among rural–urban residence with cannabis use influences (not shown in tables) showed that those in rural (vs. urban) areas reported greater coping motives (rural *M* = 4.68 [SD = 0.77], urban *M* = 4.50 [SD = 0.86]) and lower perceived addictiveness (rural *M* = 3.35 [SD= 1.98], urban *M* = 3.77 [SD = 1.94]) and harm (rural *M* = 2.39 [SD = 1.61], urban *M* = 2.84 [SD = 1.66]). A greater proportion of those reporting a mental health diagnosis (rural *N* = 230 [17%], urban *N* = 1117 [82.9%]) versus no diagnosis (rural *N* = 58 [9.4%], urban *N* = 556 [90.6%]) or physical health diagnosis (rural *N* = 51 [19.1%], urban *N* = 258 [80.9%]) versus no diagnosis (rural *N* = 227 [13.8%], urban *N* = 1415 [86.2%]) and parent cannabis use (rural *N* = 130 [16.7%], urban *N* = 647 [83.3%]) versus no use (rural *N* = 158 [13.3%], urban *N* = 1026 [86.7%]) resided in rural (vs. urban) areas (all *p*’s < 0.05).

Multivariable regressions (Table 2) indicated that rural (vs. urban) residence was associated with greater coping motives, higher odds of a mental health diagnosis, and lower harm perceptions.

### 3.3 ∣ Associations of rural–urban residence and cannabis use influences with cannabis use behaviors

Bivariate analyses (Table 1) indicated that those in rural (vs. urban) areas, those with greater coping motives, enjoyment motives, peer cannabis use, and those with (vs. without) parent use reported more frequent past-month cannabis use and past 6-month DUIC, but fewer use consequences. Greater perceived addictiveness and advertising exposure were associated with more frequent use, DUIC, and greater consequences, whereas greater perceived harm was associated with less frequent use and DUIC, but greater consequences. Those with (vs. without) a mental health diagnosis reported more frequent use and DUIC, and those with (vs. without) a physical health diagnosis reported more frequent use.

As shown in Table S2, a smaller proportion of participants living in states with (vs. without) legal non-medical cannabis, using cannabis for recreational (vs. medical) reasons, and most commonly using edibles (vs. dried herb) resided in rural (vs. urban) areas. Rural (vs. urban) participants also reported greater distance to the nearest cannabis retailer.

Multivariable regressions (Table 3) indicated that rural (vs. urban) residence was associated with more frequent cannabis use and DUIC, but fewer use consequences. When adding cannabis use influences to the model, rural (vs. urban) residence remained associated with more frequent use only. Greater coping motives, enjoyment motives, perceived addictiveness, and peer use, reporting a mental or physical health diagnosis, and reporting parent use were associated with more frequent cannabis use, and greater perceived harm was associated with less frequent use. Greater enjoyment motives, perceived addictiveness, peer use, advertising exposure, reporting a mental health diagnosis, and reporting parent use were associated with more frequent DUIC. Finally, greater perceived addictiveness and harm, advertising exposure, and reporting a physical or mental health diagnosis were associated with greater use consequences, whereas greater coping motives and peer use were associated with fewer consequences. Supplemental analyses (not shown in tables) suggested no differences in associations of cannabis use influences with use behaviors when not including rural–urban residence in models.

## 4 ∣ DISCUSSION

Consistent with hypotheses, rural- (vs. urban-) residing young adults reported greater coping motives, lower cannabis-related harm perceptions, and higher odds of a mental health diagnosis, which were associated with more frequent cannabis use and DUIC. Although prior research indicates that rural young adults may not be at greater risk for reporting any past-month cannabis use relative to urban-residing young adults,^6^ current findings align with recent research and indicate that among young adults who report any cannabis use in the past month, those in rural areas report more frequent days of use and DUIC relative to their urban counterparts.^6,7,29^ Notably, rural young adults in the current study were less likely to reside in states with legal non-medical cannabis, reported greater distance from the nearest cannabis retailer, and were less likely to use more discreet forms (e.g., edibles), suggesting that factors beyond cannabis access or form of use may be driving geographic disparities in use behaviors.

Lower cannabis-related harm perceptions may be associated with less exposure to substance use prevention messaging related to health risks in rural communities.^30,31^ Importantly, access to mental healthcare and substance use treatment is significantly lower for individuals in rural, relative to urban areas, given reduced access to providers, limited availability of specialty healthcare, a lack of trained providers, and greater stigma surrounding mental health and substance use problems in rural areas.^17^ This may be particularly true for individuals experiencing problems related to cannabis use, as rural individuals report greater perceptions of unwillingness to discuss cannabis use among their providers.^32^ Taken together,^18,33^ rural young adults may turn to cannabis to cope with stress and mental health symptoms in replacement of traditional services. Indeed, our findings indicate that a greater proportion of young adults reporting cannabis use for medical (vs. recreational) reasons resided in rural areas.

Inconsistent with hypotheses, rural–urban residence was not significantly associated with some individual-level (i.e., perceived addictiveness, enjoyment motives, physical health diagnosis, parental cannabis use) or any interpersonal (i.e., peer use) or contextual-level factors (i.e., advertising exposure) in multivariable models. Findings may suggest that rural young adults may be using cannabis as a means of coping with stress and mental health, combined with having lower perceived harm of cannabis, rather than factors related to entertainment, socialization, or advertising exposure. Also inconsistent with hypotheses, rurality was associated with lower levels of cannabis use consequences. Cannabis use consequences were assessed with items related to experiencing interpersonal disapproval and experiencing physical or mental health consequences due to cannabis use. Because rural areas are characterized by greater substance use social norms,^14,15^ it is possible that those in rural areas may be less likely to experience interpersonal disapproval related to their cannabis use. Moreover, those who use cannabis more frequently may develop a greater tolerance to the effects of cannabis and perceive fewer physical or mental health consequences,^34^ though this should be directly explored in future research.

Findings have implications for future research and public health. Researchers should consider the geographic context within which young adults reside when examining cannabis use behaviors and their correlates. It remains imperative for future researchers to use longitudinal data to examine whether cannabis use influences formally mediate associations of rural–urban residence with use behaviors. With future replication and use of longitudinal data, findings may highlight the need for public health interventions to focus on reducing problematic cannabis use behaviors among young adults within rural communities by educating young adults on healthy strategies for coping with stress and mental health symptoms, as well as the potential harms associated with frequent cannabis use and DUIC.

### 4.1 ∣ Limitations

Findings should be interpreted in light of a few limitations, including potentially limited generalizability of the sample given the use of Facebook for recruitment, ~60% of participants reporting female sex and identifying as White, and ~85% residing in urban areas; the use of cross-sectional data; reliance on a single geographic coding system; focusing on cannabis use in isolation from other substance use; and measures not being exhaustively inclusive. Findings may not be reflective of all US young adults who use cannabis and estimates should not be interpreted as prevalence rates. Future research using representative samples, longitudinal data to identify the direction of associations and potential bidirectional associations, alternative geographic classification systems, purposively recruiting to obtain a greater proportion of rural residents, examining geographic differences in cannabis along with other types of comorbid substance use, and including additional measures (e.g., cannabis potency/concentration, residential movement, addiction treatment/access) is needed.

## 5 ∣ CONCLUSIONS

Findings indicate that rural-residing young adults may engage in more frequent past-month cannabis use and DUIC, relative to their urban-residing peers. A one-size-fits-all approach to public health efforts aimed at reducing problematic cannabis use may be insufficient. Such efforts should be tailored to geographic context, and the specific factors associated with geographic differences in use behaviors, including harm perceptions, coping motives, and mental health symptoms.

## Supplementary Material

Supplemental Materials

Additional supporting information can be found online in the Supporting Information section at the end of this article.

## Figures and Tables

**TABLE 1:** Participant characteristics and bivariate associations of sociodemographic covariates, rural–urban residence, and cannabis use influences with cannabis use behaviors among participants reporting past-month cannabis use, *N* = 1961.

Variables	Total *N* = 1961 (100.0%) *M* (SD) or *N* (%)	Past-month cannabis use frequency *M* = 14.19 (SD = 11.30)		DUIC *M* = 0.91 (SD = 1.42)		Cannabis use consequences *M* = 1.99 (SD =0.86)	
		*M* (SD) or *r*	*p*	*M* (SD) or *r*	*p*	*M* (SD) or *r*	*p*
*Covariates*							
State cannabis legalization			.247		**<.001**		**<.001**
Not legal	966 (49.3)	14.49 (11.36)		1.03 (1.48)		1.92 (0.84)	
Legal	995 (50.7)	13.90 (11.25)		0.79 (1.34)		2.06 (0.87)	
Age	26.86 (4.61)	0.13	**<.001**	0.11	**<.001**	−0.13	**<.001**
Sex at birth			.316		**.003**		**<.001**
Female	1153 (59.2)	14.43 (11.37)		0.83 (1.38)		1.91 (0.82)	
Male	796 (40.8)	13.91 (11.20)		1.03 (1.46)		2.10 (0.90)	
Race			**<.001**		**<.001**		**<.001**
White	1210 (61.8)	14.69 (11.44)^a^		0.91 (1.44)^a^		1.95 (0.84)^a^	
Black	355 (18.1)	16.10 (10.93)^a^		1.21 (1.49)^b^		1.92 (0.91)^a^	
Asian	169 (8.6)	7.04 (7.86)^b^		0.41 (.95)^c^		2.37 (0.84)^b^	
Another race	224 (11.4)	13.84 (11.36)^a^		0.80 (1.32)^a^		2.03 (0.80)^a^	
Ethnicity			.488		.334		.079
Non-Hispanic	1516 (78.4)	14.34 (11.39)		0.93 (1.44)		1.97 (0.84)	
Hispanic	418 (21.6)	13.91 (11.01)		0.86 (1.35)		2.06 (0.91)	

*Rural–urban residence*			**<.001**		**.039**		**<.001**
Urban	1673 (85.3)	13.81 (11.27)		0.88 (1.39)		2.02 (0.86)	
Rural	288 (14.7)	16.44 (11.25)		1.07 (1.54)		1.79 (0.80)	

*Cannabis use influences*							
Coping motives	4.52 (0.85)	0.35	**<.001**	0.15	**<.001**	−0.17	**<.001**
Enjoyment motives	4.05 (1.11)	0.36	**<.001**	0.19	**<.001**	−0.07	**.003**
Perceived addictiveness	3.71 (1.95)	0.07	**.002**	0.09	<**.001**	0.29	<**.001**
Perceived harm	2.77 (1.66)	−0.23	**<.001**	−0.05	**.044**	0.45	**<.001**
Mental health diagnosis			**<.001**		**<.001**		.071
No	614 (31.3)	11.74 (10.60)		0.73 (1.25)		1.94 (0.83)	
Yes	1247 (68.7)	15.31 (11.44)		0.99 (1.48)		2.01 (0.87)	
Physical health diagnosis			**<.001**		.078		.853
No	1642 (83.7)	13.75 (11.21)		0.88 (1.39)		1.99 (0.86)	
Yes	319 (16.3)	16.49 (11.51)		1.05 (1.53)		1.98 (0.86)	
Peer use	3.12 (1.53)	0.35	**<.001**	0.19	**<.001**	−0.15	**<.001**
Parent use			**<.001**		**<.001**		**.013**
No	1184 (60.4)	12.34 (10.82)		0.69 (1.24)		2.03 (0.85)	
Yes	777 (39.6)	17.02 (11.44)		1.25 (1.58)		1.93 (0.88)	
Cannabis advertising exposure	1.44 (1.13)	0.12	**<.001**	0.18	**<.001**	0.13	**<.001**

*Note*: Participants who prefer not to answer across variables included *n* = 12 for sex, *n* = 3 for race, *n* = 27 for ethnicity, and *n* = 27 for DUIC. Motives assessed on a scale from 1 = Not at all to 5 = Very much; Perceived addictiveness and harm assessed on a scale from 1 = Not at all to 7 = Extremely; Peer use assessed on a scale from 0 peers to 5+ peers; Cannabis advertising exposure assessed on a scale from 1 = Not at all to 5 = More than once a day (aggregated across source); Past-month cannabis use frequency assessed on a scale from 1 day to 30 days; DUIC assessed on a scale from 0 = 0 times to 4 = 6+ times; Cannabis use consequences assessed on a scale from 1 = Not at all to 5 = Very much. Bolded values denote statistical significance at *p* < .05. Different superscripts denote statistically significant post hoc differences at *p* < .05.

Abbreviations: DUIC, driving under the influence of cannabis; SD, standard deviation.

**TABLE 2:** Multivariable regressions examining associations of sociodemographic covariates and rural–urban residence with cannabis use influences, *N* = 1961.

Variables	Coping motives *B* (SE)	Enjoyment motives *B* (SE)	Perceived addictiveness *B* (SE)	Perceived harm *B* (SE)	Mental health diagnosis aOR (95% CI)	Physical health diagnosis aOR (95% CI)	Peer use *B* (SE)	Parent use aOR (95% CI)	Advertising exposure *B* (SE)
*Covariates*									
State cannabis legalization									
Not legal	REF	REF	REF	REF	REF	REF	REF	REF	REF
Legal	−0.07 (0.04)	−0.01 (0.05)	**0.30 (0.09)**	**0.17 (0.08)**	0.84 (0.68, 1.03)	**0.69 (0.54, 0.89)**	0.13 (0.07)	**0.79 (0.65, 0.95)**	**0.29 (0.05)**
Age	**0.02 (0.01)**	**0.01 (0.01)**	−0.02 (0.01)	**−0.03 (0.01)**	0.99 (0.97, 1.01)	**1.03 (1.01, 1.06)**	**0.04 (0.01)**	1.00 (0.98, 1.02)	0.01 (0.01)
Sex at birth									
Female	REF	REF	REF	REF	REF	REF	REF	REF	REF
Male	**−0.15 (0.04)**	0.06 (0.05)	0.09 (0.09)	0.01 (0.08)	**0.49 (0.40, 0.60)**	**0.70 (0.54, 0.91)**	−0.02 (0.07)	**0.70 (0.57, 0.84)**	0.02 (0.05)
Race									
White	REF	REF	REF	REF	REF	REF	REF	REF	REF
Black	−0.01 (0.05)	**0.02 (0.07)**	**0.65 (0.12)**	0.12 (0.10)	**0.51 (0.39, 0.66)**	**0.57 (0.39, 0.82)**	0.03 (0.09)	1.24 (0.97, 1.58)	0.12 (0.07)
Asian	**−0.44 (0.07)**	**−0.37 (0.09)**	−0.01 (0.17)	**0.77 (0.14)**	**0.26 (0.19, 0.38)**	**0.55 (0.31, 0.97)**	**−0.75 (0.13)**	**0.26 (0.16, 0.41)**	−0.18 (0.10)
Another race	−0.04 (0.06)	**−0.04 (0.08)**	0.19 (0.15)	0.04 (0.13)	0.96 (0.68, 1.34)	1.27 (0.87, 1.86)	−0.06 (0.12)	0.91 (0.66, 1.24)	−0.14 (0.09)
Ethnicity									
Non-Hispanic	REF	REF	REF	REF	REF	REF	REF	REF	REF
Hispanic	−0.07 (0.05)	−0.07 (0.06)	0.17 (0.11)	**0.28 (0.10)**	**0.55 (0.43, 0.71)**	0.83 (0.60, 1.14)	**−0.30 (0.09)**	**0.77 (0.60, 0.97)**	0.05 (0.07)

*Rural–urban residence*									
Urban	REF	REF	REF	REF	REF	REF	REF	REF	REF
Rural	**0.18 (0.05)**	−0.04 (0.07)	−0.22 (0.13)	**−0.25 (0.11)**	**1.40 (1.01, 1.94)**	1.13 (0.81, 1.57)	−0.03 (0.10)	1.13 (0.81, 1.57)	0.02 (0.07)
Adjusted or Nagelkerke *R*^2^	**.049**	**.010**	**.026**	**.034**	**.118**	**.193**	**.033**	**.063**	**.017**

*Note*: Bolded values denote statistical significance at *p* < .05. REF refers to the referent group for each categorical predictor in the model.

Abbreviations: aOR, adjusted odds ratio; CI, confidence interval; SE, standard error.

**TABLE 3:** Multivariable regressions examining associations of sociodemographic covariates, rural–urban residence, and cannabis use influences on cannabis use behaviors, *N* = 1961.

Variables	Past-month cannabis use frequency		DUIC		Cannabis use consequences	
	*B* (SE)	*p*	*B* (SE)	*p*	*B* (SE)	*p*
**Step 1**						
*Covariates*						
State cannabis legalization						
Not legal	REF	REF	REF	REF	REF	REF
Legal	0.28 (0.51)	.589	**−0.20 (0.07)**	**.002**	0.09 (0.04)	**.025**
Age	0.26 (0.06)	**<.001**	0.03 (0.01)	**<.001**	−0.02 (0.01)	**<.001**
Sex at birth						
Female	REF	REF	REF	REF	REF	REF
Male	−0.27 (0.06)	.605	0.22 (0.07)	**<.001**	0.17 (0.04)	**<.001**
Race						
White	REF	REF	REF	REF	REF	REF
Black	1.74 (0.68)	**.010**	0.32 (0.09)	**<.001**	−0.07 (0.05)	.177
Asian	−6.99 (0.94)	**<.001**	−0.45 (0.12)	**<.001**	0.31 (0.07)	**<.001**
Another race	−0.36 (0.85)	.667	−0.04 (0.11)	.714	0.04 (0.06)	.562
Ethnicity						
Non-Hispanic	REF	REF	REF	REF	REF	REF
Hispanic	−0.47 (0.64)	.463	−0.04 (0.08)	.641	0.06 (0.05)	.249
*Rural–urban residence*						
Urban	REF	REF	REF	REF	REF	REF
Rural	1.90 (0.73)	**.009**	0.19 (0.09)	**.039**	−0.15 (0.06)	**.009**
Adjusted *R*^2^	.053		.036		0.049	

**Step 2**						
*Rural–urban residence*						
Urban	REF	REF	REF	REF	REF	REF
Rural	1.57 (0.64)	**.014**	0.12 (0.09)	.176	−0.10 (0.05)	.050
*Cannabis use influences*						
Coping motives	2.10 (0.30)	**<.001**	0.05 (0.04)	.224	−0.09 (0.02)	**<.001**
Enjoyment motives	1.85 ( 0.23)	**<.001**	0.12 (0.03)	**<.001**	0.01 (0.02)	.736
Perceived addictiveness	0.49 (0.12)	**<.001**	0.05 (0.02)	**.005**	0.07 (0.10)	**<.001**
Perceived harm	−0.91 (0.15)	**<.001**	−0.01 (0.02)	.969	0.18 (0.01)	**<.001**
Mental health diagnosis						
No	REF	REF	REF	REF	REF	REF
Yes	1.74 (0.50)	**<.001**	0.17 (0.07)	**.018**	0.21 (0.04)	**<.001**
Physical health diagnosis						
No	REF	REF	REF	REF	REF	REF
Yes	1.24 (0.61)	**.043**	0.08 (0.09)	.354	0.09 (0.05)	**.049**
Peer use	1.49 (0.16)	**<.001**	0.08 (0.02)	**<.001**	−0.05 (0.01)	**<.001**
Parent use						
No	REF	REF	REF	REF	REF	REF
Yes	1.51 (0.47)	**.001**	0.37 (0.07)	**<.001**	0.04 (0.04)	.230
Cannabis advertising exposure	0.25 (0.20)	.226	0.17 (0.03)	**<.001**	0.07 (0.02)	**<.001**
Adjusted *R*^2^	.276		.123		.275	

*Note*: Bolded values denote statistical significance at *p* < .05. Findings for covariates in relation to outcomes were consistent across Steps 1 and 2 and thus, are reported for Step 1 only. REF refers to the referent group for each categorical predictor in the model.

Abbreviations: DUIC, driving under the influence of cannabis; SE, standard error.

## Data Availability

The data that support the findings of this study are available from the corresponding author upon reasonable request.
