# Supplementary material for: Rural–urban residence in relation to cannabis use influences and behaviors among a sample of US young adults reporting past-month cannabis use
Source: Am J Addict. Author manuscript; Available in PMC 2026 Aug 24. (PMC13485314; doi:10.1111/ajad.70185)
Supplement: Supplementary file 1 [file NIHMS2201324-supplement-Supplemental_Materials.docx]

| **Supplemental Table 1. Cannabis legalization, geographic region, and proportion of participants in rural vs. urban areas by state, N=1,961** | | | | | |
| --- | --- | --- | --- | --- | --- |
| **State** | **Total Participants,**  **N=1,961 (100.0)** | **Rural Participants**  **N=288 (14.7)** | **Urban Participants N=1,673 (85.3)** | **Cannabis Legalization Status** | **CDC Geographic Region** |
|  | **N (%)** | **N (%)** | **N (%)** |  |  |
| AL | 20 (1.0) | 3 (1.0) | 17 (1.0) | Legal for medical use | South |
| AK | 1 (0.1) | 0 (0.0) | 1 (0.1) | Legal for non-medical use | West |
| AZ | 56 (2.9) | 4 (1.4) | 52 (3.1) | Legal for non-medical use | West |
| AR | 19 (1.0) | 8 (2.8) | 11 (0.7) | Legal for medical use | South |
| CA | 240 (12.2) | 10 (3.5) | 230 (13.7) | Legal for non-medical use | West |
| CO | 29 (1.5) | 6 (2.1) | 23 (1.4) | Legal for non-medical use | West |
| CT | 15 (0.8) | 0 (0.0) | 15 (0.9) | Legal for non-medical use | Northeast |
| DE | 7 (0.4) | 2 (0.7) | 5 (0.3) | Legal for non-medical use | South |
| DC | 10 (0.5) | 0 (0.0) | 10 (0.6) | Legal for non-medical use | South |
| FL | 112 (5.7) | 8 (2.8) | 104 (6.2) | Legal for medical use | South |
| GA | 51 (2.6) | 6 (2.1) | 45 (2.7) | Legal for medical use | South |
| HI | 2 (0.1) | 0 (0.0) | 2 (0.1) | Legal for medical use | West |
| ID | 2 (0.1) | 0 (0.0) | 2 (0.1) | Not legal | West |
| IL | 82 (4.2) | 10 (3.5) | 72 (4.3) | Legal for non-medical use | Midwest |
| IN | 31 (1.6) | 8 (2.8) | 23 (1.4) | Not legal | Midwest |
| IA | 12 (0.6) | 6 (2.1) | 6 (0.4)6 (0.4) | Not legal | Midwest |
| KS | 14 (0.7) | 4 (1.3) | 10 (0.6) | Not legal | Midwest |
| KY | 39 (2.0) | 13 (4.5) | 26 (1.6) | Legal for medical use | South |
| LA | 22 (1.1) | 0 (0.0) | 22 (1.3) | Legal for medical use | South |
| ME | 10 (0.5) | 6 (2.1) | 4 (0.2) | Legal for non-medical use | Northeast |
| MD | 39 (2.0) | 2 (0.7) | 37 (2.2) | Legal for non-medical use | South |
| MA | 34 (1.7) | 1 (0.3) | 33 (2.0) | Legal for non-medical use | Northeast |
| MI | 72 (3.7) | 9 (3.1) | 63 (3.8) | Legal for non-medical use | Midwest |
| MN | 25 (1.3) | 5 (1.7) | 20 (1.2) | Legal for non-medical use | Midwest |
| MS | 25 (1.3) | 14 (4.9) | 11 (0.7) | Legal for medical use | South |
| MO | 57 (2.9) | 13 (4.5) | 44 (2.6) | Legal for non-medical use | Midwest |
| MT | 7 (0.4) | 5 (1.7) | 2 (0.1) | Legal for non-medical use | West |
| NE | 9 (0.5) | 1 (0.3) | 8 (0.5) | Legal for medical use | Midwest |
| NV | 32 (1.6) | 4 (1.4) | 28 (1.7) | Legal for non-medical use | West |
| NH | 3 (0.2) | 2 (0.7) | 1 (0.1) | Legal for medical use | Northeast |
| NJ | 37 (1.9) | 0 (0.0) | 37 (2.2) | Legal for non-medical use | Northeast |
| NM | 6 (0.3) | 1 (0.3) | 5 (0.3) | Legal for non-medical use | West |
| NY | 168 (8.6) | 10 (3.5) | 158 (9.4) | Legal for non-medical use | Northeast |
| NC | 55 (2.8) | 14 (4.9) | 41 (2.5) | Not legal | South |
| ND | 0 (0.0) | 0 (0.0) | 0 (0.0) | Legal for medical use | Midwest |
| OH | 86 (4.4) | 20 (6.9) | 66 (3.9) | Legal for non-medical use | Midwest |
| OK | 33 (1.7) | 13 (4.5) | 20 (1.2) | Legal for medical use | South |
| OR | 29 (1.5) | 9 (3.1) | 20 (1.2) | Legal for non-medical use | West |
| PA | 82 (4.2) | 8 (2.8) | 74 (4.4) | Legal for medical use | Northeast |
| RI | 11 (0.6) | 0 (0.0) | 11 (0.7) | Legal for non-medical use | Northeast |
| SC | 29 (1.5) | 9 (3.1) | 20 (1.2) | Not legal | South |
| SD | 3 (0.2) | 1 (0.3) | 2 (0.1) | Legal for medical use | Midwest |
| TN | 36 (1.8) | 10 (3.5) | 26 (1.6) | Not legal | South |
| TX | 160 (8.2) | 19 (6.6) | 141 (8.4) | Legal for medical use | South |
| UT | 15 (0.8) | 1 (0.3) | 14 (0.8) | Legal for medical use | West |
| VT | 5 (0.3) | 4 (1.4) | 1 (0.1) | Legal for non-medical use | Northeast |
| VA | 48 (2.4) | 8 (2.8) | 40 (2.4) | Legal for non-medical use | South |
| WA | 36 (1.8) | 1 (0.3) | 35 (2.1) | Legal for non-medical use | West |
| WV | 18 (0.9) | 5 (1.7) | 13 (0.8) | Legal for medical use | South |
| WI | 26 (1.3) | 4 (1.4) | 22 (1.3) | Not legal | Midwest |
| WY | 1 (0.1) | 1 (0.3) | 0 (0.0) | Not legal | West |

| **Supplemental Table 2. Associations of rural-urban residence with cannabis use characteristics among participants reporting past-month cannabis use, N=1,961** | | | | | | | | | | | | | |
| --- | --- | --- | --- | --- | --- | --- | --- | --- | --- | --- | --- | --- | --- |
|  | **State Non-Medical Cannabis Legalization** | | | **Distance to Cannabis Retailer(s)** | | **Most Common Mode of Cannabis Use** | | | | | **Medical/Recreational Use** | | |
|  | **Not Legal N=966 (49.3)** | **Legal N=995 (50.7)** |  | **M=3.16**  **(SD=1.89)** | | **Dried Herb N=1098 (56.3)** | **Oils N=281 (14.4)** | **Concentrates/**  **Other N=172 (8.8)** | **Edibles N=398 (20.4)** |  | **Medical Use N=648 (35.0)** | **Recreational Use N=1206 (65.0)** |  |
| **Variables** | **N (%)** | **N (%)** | **p** | **M (SD) or r** | **p** | **N (%)** | **N (%)** | **N (%)** | **N (%)** | **p** | **N (%)** | **N (%)** | **p** |
| *Rural-Urban Residence* | |  | **<.001** |  | **<.001** |  |  |  |  | **.012** |  |  | **<.001** |
| Urban | **772 (79.9)^a^** | **901 (90.6)^b^** |  | **3.05 (1.80)** |  | **919 (83.7)^a^** | **240 (85.4)^a,b^** | **144 (83.7)^a,b^** | **360 (90.5)^b^** |  | **523 (80.7)^a^** | **1057 (87.6)^b^** |  |
| Rural | **194 (20.1)^a^** | **94 (9.4)^b^** |  | **3.86 (2.24)** |  | **179 (16.3)^a^** | **41 (14.6)^a,b^** | **28 (16.3)^a,b^** | **38 (9.5)^b^** |  | **125 (19.3)^a^** | **149 (12.4)^b^** |  |
| *Note:* Participants reporting prefer not to answer or don't know across variables included n=273 for distance to cannabis retailer, N=12 for mode of use, and N=107 for medical/recreational use. Distance to cannabis retailer assessed on a scale from 1=Less than 5 minutes to 8=More than one hour. Bolded values denote statistical significance at p<.05. Different superscripts denote statistically significant post-hoc differences at p<.05. | | | | | | | | | | | | | |
